# Durable partial response with low-dose, intermittent nivolumab in combination with gemcitabine for high PD-L1 metastatic triple-negative breast cancer: a case report

**DOI:** 10.3389/fonc.2026.1964934

**Published:** 2026-10-09

**Authors:** Po Lin Ooi, Jamin Munchar Elias, Francis Yew Fu Tieng

**Affiliations:** 1 Department of Clinical Oncology, Faculty of Medicine, Universiti Malaya, Jalan Universiti, Kuala Lumpur, Malaysia; 2 Department of Clinical Oncology, Universiti Malaya Medical Centre, Jalan Profesor Diraja Ungku Aziz, Petaling Jaya, Malaysia; 3 Center of Excellence for Innovative Medical Devices, University Malaya, Kuala Lumpur, Malaysia

**Keywords:** combination therapy, dose de-escalation, gemcitabine, immune checkpoint inhibitors, nivolumab, PD-L1, triple-negative breast cancer

## Abstract

Immune checkpoint inhibitors (ICIs) have improved outcomes in selected patients with metastatic triple-negative breast cancer (mTNBC), particularly those with high programed death-ligand 1 (PD-L1) expression. However, optimal dosing strategies balancing efficacy, toxicity, and cost remain unclear. We report the case of a 34-year-old woman with recurrent mTNBC and high PD-L1 expression (combined positive score, CPS ≥ 60; immunohistochemistry 22C3 pharmDx assay). She achieved durable disease control over 22 months (September 2024 to June 2026) using a combination of gemcitabine maintenance chemotherapy and low-dose intermittent nivolumab (40 mg fixed dose, administered 3-weekly). This de-escalated regimen was adopted due to early treatment toxicity and severe financial constraints. The patient achieved a confirmed partial response by RECIST v1.1 criteria (48% reduction in target lesion size), accompanied by manageable CTCAE Grade 2 hypothyroidism requiring oral levothyroxine replacement. This case illustrates that in biomarker-selected, PD-L1-high mTNBC, combination chemotherapy with low-intensity PD-1 blockade can maintain prolonged antitumor activity. These observations support the working hypothesis of a minimum effective immune dose, warranting prospective evaluation in resource-constrained clinical settings.

## Highlights

- A 34-year-old woman with mTNBC and high PD-L1 expression (CPS ≥ 60) achieved a durable partial response—including more than a 30% reduction in a chest wall lesion—using gemcitabine and low-dose, intermittent nivolumab (40 mg fixed dose).
- Disease control was sustained over a 22-month follow-up period (September 2024 to June 2026) despite a substantially lower cumulative nivolumab dose compared to standard regimens.
- The favorable clinical outcome reflects the combined synergistic efficacy of cytotoxic gemcitabine chemotherapy and low-dose PD-1 blockade in a biomarker-selected, PD-L1-positive tumor microenvironment.
- The low-dose strategy was implemented due to initial treatment toxicity and severe financial constraints, highlighting a pragmatic, cost-effective approach for resource-limited healthcare settings.

## 1 Introduction

Triple-negative breast cancer (TNBC) is an aggressive subtype accounting for approximately 15% of breast cancers and is associated with inferior outcomes in the metastatic setting (1). The introduction of immune checkpoint inhibitors (ICI) targeting the PD-1/PD-L1 axis, such as Pembrolizumab and Atezolizumab, has expanded treatment options for patients with PD-L1–positive disease. In the KEYNOTE-355 trial, the addition of pembrolizumab to chemotherapy significantly improved progression-free survival in patients with PD-L1–positive metastatic TNBC (combined positive score ≥10), with median progression-free survival of 9.7 months versus 5.6 months with chemotherapy alone. However, durable clinical benefit—defined as sustained objective response or prolonged progression-free survival (PFS)—remains limited to a subset of patients, even among those with PD-L1–positive tumors (2). Furthermore, currently approved dosing schedules for PD-1 inhibitors, including nivolumab and pembrolizumab, were largely established during early phase clinical development and are not based on a clearly defined pharmacodynamic dose–response relationship (3).

Emerging pharmacokinetic and pharmacodynamic data suggest that PD-1 receptor occupancy may be achieved at doses substantially lower than those routinely administered in clinical practice. Early nivolumab trials showed antitumor activity across multiple dose levels, including the lowest doses tested, without a clear dose–response relationship in efficacy (4, 5). Supporting this concept, Sun et al. demonstrated that reduced-dose PD-1 blockade, administered either as monotherapy or in combination with chemotherapy, maintained meaningful clinical activity while exhibiting an acceptable safety profile (6). In addition, pharmacokinetic analysis of Nivolumab have demonstrated a relatively flat exposure–response relationship across dose ranges, suggesting that maximal biological activity may occur at lower doses than those used in standard regimens (7). These observations raise important questions regarding dose optimization, particularly in real-world settings where toxicity, treatment interruptions, and global financial barriers frequently influence care delivery.

Here, we describe a case report of a patient with metastatic TNBC and exceptionally high PD-L1 expression who achieved durable disease control over 22 months (September 2024 to June 2026) when treated with a combination of gemcitabine chemotherapy and low-dose, intermittent nivolumab (40 mg fixed dose, administered 3-weekly).

## 2 Case presentation

A 34-year-old woman presented in early 2023 with a palpable right breast mass and ipsilateral axillary lymphadenopathy. Core needle biopsy confirmed triple-negative breast carcinoma. Initial staging was cT3N1M0.

The patient received neoadjuvant chemotherapy with three cycles of epirubicin and cyclophosphamide followed by three cycles of paclitaxel (EC ×3 → T ×3), which was completed in May 2023. In June 2023, she underwent a right modified radical mastectomy with axillary lymph node dissection. Pathologic examination demonstrated residual invasive carcinoma, Bloom-Richardson grade 3, with a final pathological stage of ypT2N0. Surgical margins were negative, and no lymphovascular or perineural invasion was identified. Immunohistochemistry (IHC) confirmed a triple-negative phenotype with estrogen receptor expression <1% (weak staining), progesterone receptor negativity, and HER2 negativity.

The patient subsequently received adjuvant radiotherapy to the right chest wall and supraclavicular fossa (40 Gy in 15 fractions) in September 2023. This was followed by eight cycles of adjuvant capecitabine, completed in March 2024. Shortly after completion of adjuvant capecitabine, she developed locoregional recurrence involving the chest wall with concurrent pulmonary metastases, consistent with metastatic disease progression.

First-line palliative chemotherapy with paclitaxel and carboplatin was administered for five cycles. However, interval imaging demonstrated disease progression with worsening lung and left hilar nodal metastases. Biomarker testing of the recurrent chest wall biopsy tissue was conducted using PD-L1 immunohistochemistry (22C3 pharmDx assay on the Dako Autostainer Link 48 platform), demonstrating an exceptionally high CPS ≥ 60. Due to diagnostic resource limitations and severe financial constraints, additional molecular profiling—such as tumor mutational burden (TMB), next-generation genomic sequencing, or quantitative tumor-infiltrating lymphocyte (TIL) scoring—was not performed.

Second-line combination therapy was initiated in September 2024 with gemcitabine (1,000 mg/m² on days 1 and 8), carboplatin (AUC 2 on days 1 and 8), and fixed-dose low-intensity nivolumab (40 mg on day 1 of a 3-week cycle). The 40 mg fixed dose (approximately 17% of the standard 240 mg biweekly dose) was selected due to severe financial toxicity, as full-dose immunotherapy was unaffordable. Following two initial cycles, treatment was complicated by CTCAE Grade 3 thrombocytopenia and Grade 2 transaminitis, necessitating treatment delays and omission of day-8 gemcitabine. Carboplatin was subsequently discontinued, and the patient transitioned to gemcitabine maintenance chemotherapy (1,000 mg/m² on day 1 every 3 weeks) in combination with intermittent fixed-dose nivolumab (40 mg on day 1 every 3 weeks).

Serial CT imaging provided systematic documentation of target lesions according to RECIST v1.1 criteria. Baseline CT imaging (September 2024) demonstrated a prominent right chest wall target mass measuring 2.5 cm × 4.8 cm × 3.7 cm with skin ulceration, alongside bilateral pulmonary nodules, and hilar lymphadenopathy (**Figure 1A**). Follow-up CT imaging confirmed a partial response, demonstrating a 48% reduction in the sum of longest diameters of the target chest wall lesion (measuring 1.3 cm × 2.5 cm × 2.5 cm) alongside stable bilateral pulmonary nodules and regression of hilar lymphadenopathy (**Figure 1B**). Clinically, the chest wall ulceration completely re-epithelialized into a dry, stable scar.

**Figure 1:**
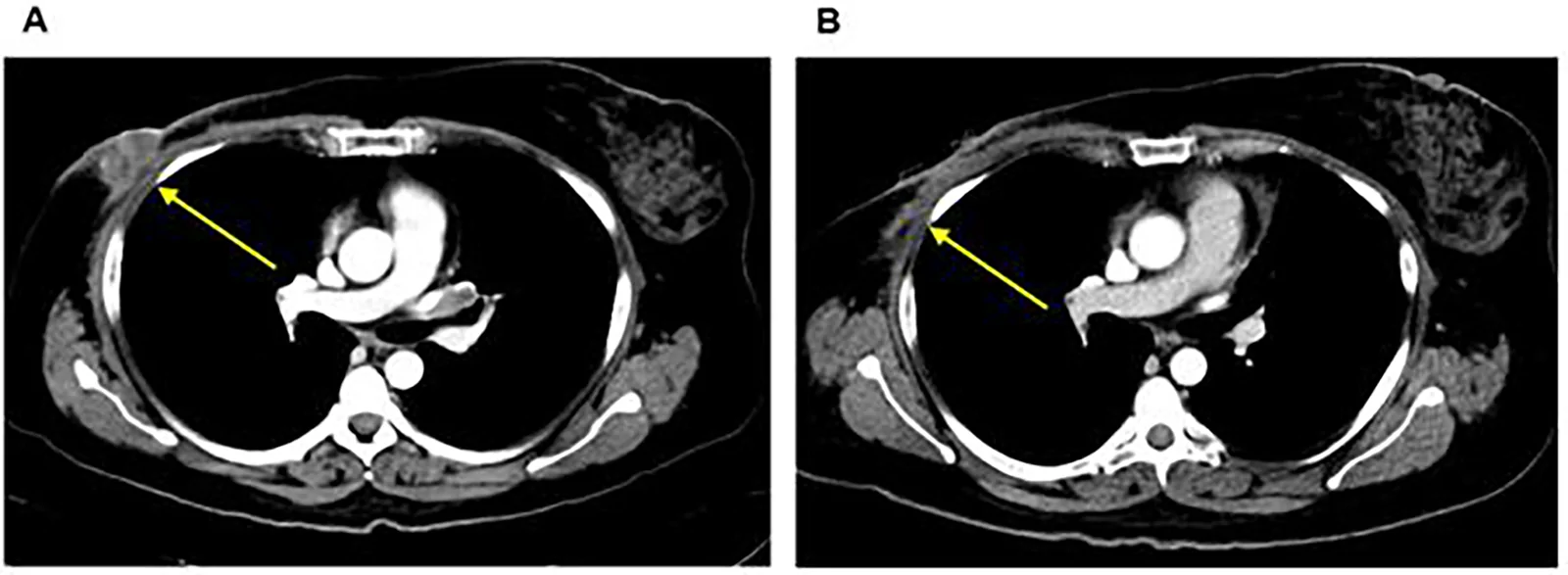
Computed tomography of the thorax. **(A)** Baseline imaging demonstrating a prominent right chest wall mass consistent with locoregional recurrence measuring 2.5cm X 4.8cm X 3.7cm. **(B)** Follow-up imaging demonstrating an interval reduction of the right chest wall mass, now measuring 1.3cm x 2.5cm x 2.5cm, consistent with ongoing disease control during low-dose nivolumab maintenance.

Regarding safety, the patient developed CTCAE Grade 2 immune-related hypothyroidism after 6 months of therapy, presenting with mild fatigue and elevated thyroid-stimulating hormone (TSH > 10 mIU/L). This was successfully managed with oral levothyroxine (150 µg daily) under endocrinological follow-up without interrupting cancer treatment. No Grade 3 or 4 immune-related adverse events occurred. The patient maintained continuous therapy through Phase 4 (September 2025 to June 2026), receiving 3-weekly gemcitabine maintenance and low-dose nivolumab (40 mg on Day 1). Over the 22-month treatment timeline (September 2024 to June 2026), she received 27 total doses of low-dose nivolumab 40 mg (cumulative dose: 1,080 mg) and maintained an ECOG performance status of 0 to 1 with durable disease control (**Figure 2**).

**Figure 2:**
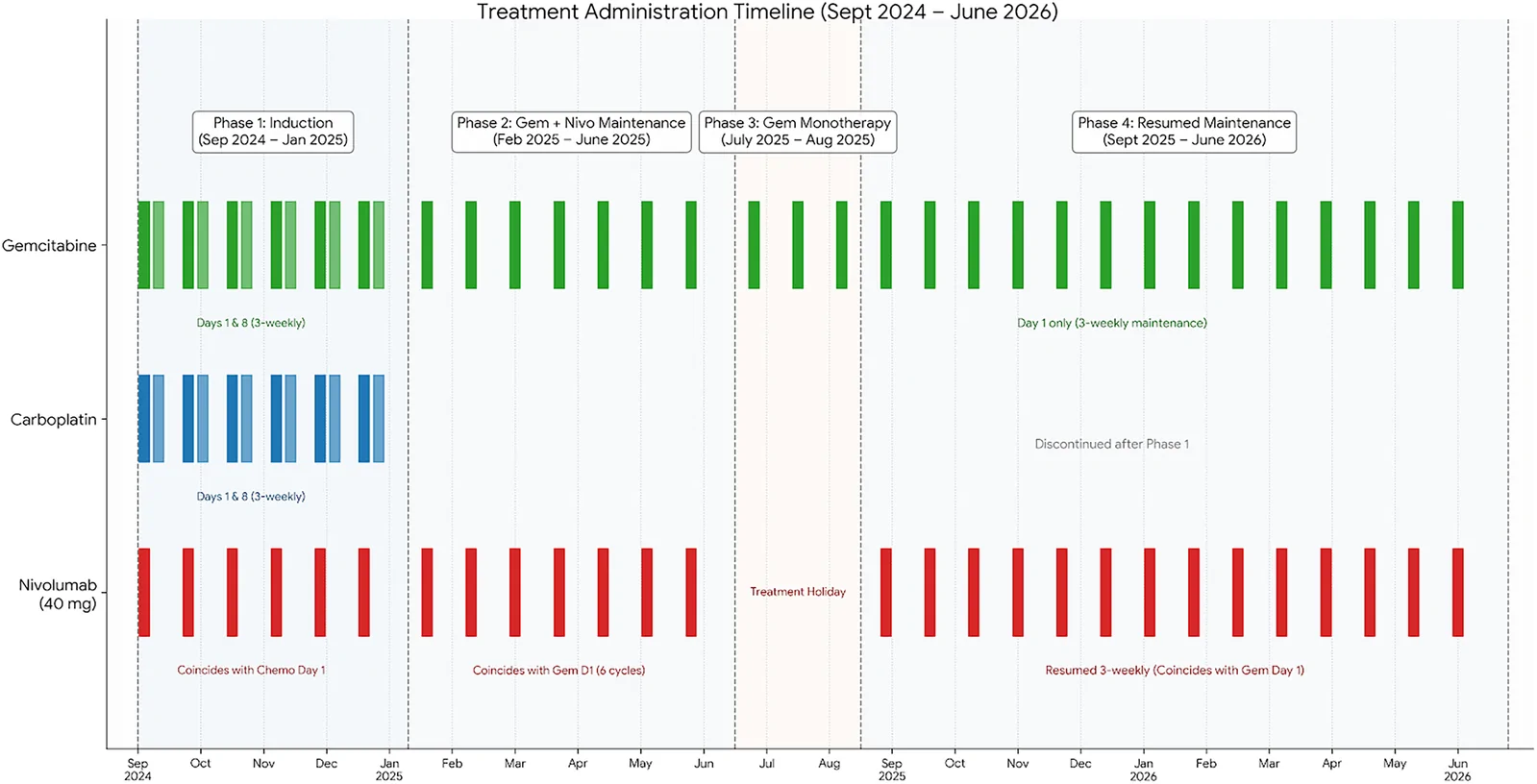
Clinical treatment timeline (September 2024 – June 2026). Overview of second-line chemo-immunotherapy administration. Phase 1 (induction): 3-weekly gemcitabine (Days 1, 8), carboplatin (Days 1, 8), and low-dose nivolumab 40 mg (Day 1). Phase 2 (gemcitabine maintenance + nivolumab): transition to 3-weekly gemcitabine (Day 1) with low-dose nivolumab 40 mg (Day 1) for 6 cycles. Phase 3 (gemcitabine monotherapy): gemcitabine maintenance (Day 1) with nivolumab treatment holiday. Phase 4 (resumed maintenance): Resumed 3-weekly gemcitabine (Day 1) and low-dose nivolumab 40 mg (Day 1) through June 2026, sustaining long-term disease control.

## 3 Discussions

Metastatic TNBC (mTNBC) is characterized by an aggressive clinical course, high rates of visceral metastasis, and rapid development of chemoresistance, rendering it an incurable disease with a poor prognosis (8). Because mTNBC exhibits higher genomic instability and greater levels of TILs compared to hormone receptor-positive subtypes, it is recognized as more immunogenic (9). This understanding has driven the integration of ICIs into standard-of-care regimens. While monoclonal antibodies targeting the PD-1/PD-L1 axis have revolutionized the treatment of PD-L1-positive mTNBC, the objective response rates (ORR) remain variable, and standard continuous dosing regimens are frequently limited by severe immune-related toxicities (10).

### 3.1 The selection of nivolumab

The choice of nivolumab over other ICIs like pembrolizumab was informed by both clinical evidence and financial pragmatism. Nivolumab has shown consistent activity in mTNBC as a backbone in trials like KORNELIA (ORR 28.9%) (11) and TONIC (12). However, in a real-world setting, the patient’s inability to afford other forms of immunotherapy made nivolumab 40 mg a necessary, cost-effective alternative. This case demonstrates that nivolumab remains a potent option even when de-escalated, providing a framework for managing ‘financial toxicity’ without sacrificing clinical outcomes.

### 3.2 Efficacy of nivolumab in metastatic breast cancer

Several clinical trials have evaluated the efficacy of nivolumab in advanced breast cancer, establishing important benchmarks for PFS and overall survival (OS). In the phase IB/II KORNELIA trial, which combined nivolumab with eribulin, the mTNBC cohort demonstrated an objective response rate (ORR) of 28.9%, a median PFS of 3.0 months, and a median OS of 15.7 months (11). Conversely, the HR-positive/HER2-negative cohort in the same study showed an ORR of 53.3%, a median PFS of 5.6 months, and a median OS of 17.9 months (13). More robust outcomes were observed in a phase Ib trial combining nivolumab, ipilimumab, and the histone deacetylase inhibitor entinostat. In this study, patients with mTNBC achieved a promising ORR of 40%, a median PFS of 9.6 months, and an extended median OS of 18.2 months (14).

Notably, the biological responsiveness to nivolumab extends beyond traditional TNBC. Data from the TONIC trial indicate that ER-low (ER 1–9%) advanced breast cancers behave biologically similarly to TNBC, exhibiting comparable median PFS (1.7 vs. 2.0 months) and median OS (5.3 vs. 8.6 months) when treated with nivolumab (12). Furthermore, combining nivolumab with the antibody-drug conjugate trastuzumab deruxtecan has yielded a median PFS of 7.0 months and a median OS of 19.5 months in patients with HER2-low metastatic breast cancer (mBC) (13). **Table 1** summarized clinical trials that evaluates nivolumab-containing regimens in mBC clinical trials versus the present case report.

**Table 1:** Nivolumab combination strategies in mBC versus the present case report.

Study/trial	Patient population	Regimen & dosing strategy	Efficacy outcomes	Safety & toxicity profile
KORNELIA Trial (11, 15)	HER2-negative mBC (TNBC cohort, n=38)	Nivolumab 240 mg q2w + eribulin	ORR 28.9%, median PFS 3.0 months, median OS 15.7 months	Grade ≥3 treatment-related AEs in 44.7% of patients
NEWFLAME Trial (16)	HR+/HER2- mBC (Phase II)	Nivolumab + abemaciclib + endocrine therapy	Terminated early due to toxicity; efficacy endpoints unconfirmed	Severe immune-related hepatotoxicity, fatal interstitial lung disease
ICON Trial (17)	HR+/HER2- mBC (Phase IIb)	Nivolumab + ipilimumab + anthracycline chemotherapy	Median PFS 5.1 vs 3.6 months (HR 0.94); no OS benefit	Serious adverse events in 63% of patients in immune-chemotherapy arm
DS8201-A-U105 (13)	HER2-low mBC (n = 16)	Trastuzumab deruxtecan + nivolumab	ORR 50%; median PFS 7.0 months; median OS 19.5 months	Treatment-related AEs were common
TONIC (12)	Metastatic hormone receptor-positive (HR+)/HER2-negative breast cancer	Nivolumab (anti-PD-1) mono-therapy following 2 weeks of random induction: no induction; radiation (3x8 Gy); cyclophosphamide; cisplatin; doxorubicin	Overall ORR 20%; Doxorubicin pre-treatment ORR 35%	Standard, manageable anti-PD-1 profile; short-term induction did not compromise safety or add severe systemic toxicities
Present Case Report	Recurrent mTNBC (PD-L1 CPS ≥ 60)	Low-dose nivolumab (40 mg intermittent, q3w) + gemcitabine maintenance	Confirmed RECIST v1.1 Partial Response (48% reduction), PFS >22 months	CTCAE Grade 2 hypothyroidism (managed with oral levothyroxine)

AE, adverse event; CPS, combined positive score; CTCAE, Common Terminology Criteria for Adverse Events; HER2−, human epidermal growth factor receptor 2-negative; HR+, hormone receptor-positive; ILD, interstitial lung disease; irAEs, immune-related adverse events; mBC, metastatic breast cancer; mTNBC, metastatic triple-negative breast cancer; ORR, overall response rate; OS, overall survival; PD-L1, programed death-ligand 1; PFS, progression-free survival; PR, partial response; q2w, every 2 weeks; q3w, every 3 weeks; RECIST v1.1, Response Evaluation Criteria in Solid Tumors version 1.1; TNBC, triple-negative breast cancer; TRAEs, treatment-related adverse events.

These findings highlight that while nivolumab demonstrates clinical activity across breast cancer subtypes, responses are heterogeneous and often modest in unselected populations, underscoring the need for improved patient selection and optimized treatment strategies.

### 3.3 Interpretation of clinical response: combination therapy vs. single-agent effect

A critical consideration in evaluating this case is distinguishing the therapeutic contribution of low-dose nivolumab from that of concurrent gemcitabine chemotherapy. Rather than attributing clinical outcomes exclusively to nivolumab, the observed durable partial response is best understood as the result of combination chemo-immunotherapy. Gemcitabine chemotherapy provides cytotoxic tumor debulking and induces immunogenic cell death, which promotes tumor antigen release and enhances immune recognition (6, 18). Concurrently, low-dose nivolumab provides PD-1 receptor blockade to prevent T-cell exhaustion. Thus, the 22-month disease control reflects the synergistic activity of gemcitabine cytotoxic maintenance and intermittent PD-1 inhibition.

### 3.4 The rationale for low-dose intermittent therapy

The use of a fixed 40 mg dose of nivolumab in this case represents an ‘ultra-low’ dose strategy, providing a stark contrast to the standard dosing regimens of 240 mg every two weeks or 480 mg every four weeks. While these standard doses were established during early development to ensure maximal saturation, our approach aligns with the findings of Sun et al., who demonstrated that reduced-dose PD-1 blockade retains clinical activity in advanced NSCLC (6). However, our case extends this concept to mTNBC, suggesting that a nearly 6-to-12-fold reduction in dose can still sustain a durable partial response for 22 months in a carefully selected patient.

Pharmacodynamic studies have shown that PD-1 receptor occupancy plateaus at doses as low as 0.3 mg/kg, with minimal incremental binding achieved at higher doses (19). Furthermore, low absolute doses of nivolumab (40 mg) have been reported to maintain saturated receptor occupancy for prolonged periods in advanced cancers (7, 18). These findings support the existence of a biological threshold of immune activation, beyond which further dose escalation may not meaningfully enhance antitumor efficacy. We propose that this threshold may represent a “minimum effective immune dose”.

Despite encouraging efficacy signals associated with nivolumab combinations, standard continuous dosing regimens are frequently limited by its treatment-related toxicities, making prolonged uninterrupted treatment challenging. The phase II NEWFLAME trial, which combined nivolumab with abemaciclib and endocrine therapy, was terminated early due to severe, lingering immune-related hepatotoxicity and a fatal case of interstitial lung disease. Consequently, definitive PFS and OS could not be determined (16). Similarly, the phase 2b ICON trial found that the concomitant addition of ipilimumab and nivolumab to anthracycline-based chemotherapy significantly increased the risk of serious adverse events (63% in the immune-chemotherapy arm) without improving median PFS (5.1 months vs. 3.6 months) or OS (HR 1.01) compared to chemotherapy alone (17). These findings suggest that increasing immune activation through higher or continuous dosing does not necessarily translate into improved clinical efficacy.

Against this backdrop of substantial toxicity reported with some nivolumab-containing combination regimens, our case provides hypothesis-generating real-world evidence of durable disease control with a low-dose (40 mg), intermittent nivolumab strategy combined with gemcitabine. The use of low-dose nivolumab in this case is supported by emerging pharmacokinetic data showing measurable nivolumab exposure following a 40-mg flat dose, although the clinical efficacy of this dosing strategy remains insufficiently established (7). In 2026, Mourad et al. reported that reduced ICI dose intensity (<75% of the recommended dose) was associated with similar response rates and no significant differences in PFS or OS among patients with MSI-H/dMMR gastrointestinal cancers (20). Although limited by its small, heterogeneous, retrospective cohort, the study provides supportive real-world evidence that reduced ICI dose intensity may be feasible when standard dosing is inaccessible, offering broader context for the reduced-dose approach used in the present case. The patient’s high PD-L1 expression may have contributed to treatment responsiveness. However, the contribution of PD-L1 expression, gemcitabine, and low-dose nivolumab to the observed response cannot be separated in this single case. During the 22-month follow-up, the patient experienced only Grade 2 hypothyroidism requiring hormone replacement and no Grade 3/4 irAEs. Therefore, rather than demonstrating that intermittent low-dose nivolumab prevents immune-related toxicity, this case raises the hypothesis that lower and less frequent nivolumab exposure may represent a potentially tolerable treatment approach while retaining clinical activity in selected patients, which warrants prospective pharmacokinetic, pharmacodynamic, and clinical evaluation.

### 3.5 Biomarkers of sustained response

The sustained responses observed in our patient underscore the importance of precision biomarker selection. Prolonged survival outcomes following nivolumab therapy are increasingly associated with distinct transcriptomic and cellular profiles. Genomic and transcriptomic analysis from the KORNELIA trial revealed that long responders (PFS ≥ 18 months) were characterized by a significantly higher proportion of naïve B-cells and plasma cells in the tumor microenvironment. Specifically, patients with high naïve B-cell infiltration achieved a significantly longer median PFS (5.6 months) compared to those with low infiltration (4.2 months). High enrichment of antigen presentation gene signatures was also significantly associated with longer PFS (5.6 vs. 4.2 months). Conversely, poorer PFS was correlated with high E2F cell-cycle signatures, homologous recombination deficiency (HRD), and TP53 mutations (11, 15).

Furthermore, novel vulnerabilities are being identified that may predict heightened sensitivity to nivolumab. For instance, the genetic deletion or loss of NEDD8 in TNBC cells has been shown to radically enhance immunogenicity, leading to increased release of interferon-gamma and granzyme B upon exposure to nivolumab (21). The robust responses seen in our PD-L1 high patient suggest that selecting for highly primed tumor microenvironments may negate the need for standard, high-dose continuous ICI exposure.

Together, these findings support the concept that treatment response is driven more by tumor immunobiology than by drug dose intensity alone.

### 3.6 The role of PD-L1 and the tumor microenvironment

Our patient’s high PD-L1 expression likely played a crucial role in enabling the success of a de-escalated ICI strategy. While TNBC is generally recognized as the most immunogenic breast cancer subtype due to higher rates of TILs and PD-L1 expression, responses to PD-1 blockade remain heterogeneous (10). In the KORNELIA trial, PD-L1 status alone did not significantly stratify objective response rates to the nivolumab and eribulin combination (11). However, translational data from the same trial indicate that patients who achieve highly sustained responses (PFS ≥ 18 months) possess distinctly “hot” tumor microenvironments, characterized by high enrichment of antigen presentation gene signatures and significantly higher proportions of naïve B-cells and plasma cells.

The clinical relevance of PD-L1 as a predictive biomarker in metastatic TNBC is well established. In KEYNOTE-355, the addition of pembrolizumab to chemotherapy significantly improved progression-free survival in patients with PD-L1 CPS ≥10 (9.7 vs. 5.6 months), confirming the therapeutic importance of PD-1/PD-L1 pathway inhibition in biomarker-selected disease (2). Similarly, IMpassion130 demonstrated meaningful benefit from atezolizumab-based therapy in PD-L1-positive TNBC, further validating this immune axis as a therapeutic target (22). While these landmark studies employed standard-dose, continuous immune checkpoint blockade, they did not specifically address whether patients with exceptionally high PD-L1 expression derive maximal benefit from conventional dosing intensity.

It is highly plausible that the patient’s strong baseline PD-L1 expression (CPS ≥60) reflects an immunologically primed tumor microenvironment. However, this interpretation remains a mechanistic hypothesis, as the present case did not include direct histological assessment or quantification of TILs In such a potentially inflamed tumor, a low-dose “nudge” with 40 mg of nivolumab may have been sufficient to sustain immune activation. This hypothesis is consistent with observations in tumors with novel vulnerabilities, such as NEDD8 loss, which can enhance interferon-gamma and granzyme B responses following ICI exposure (21). Our case therefore raises the hypothesis that, in a subset of patients with very high PD-L1 expression and potentially primed immune microenvironments, durable immune control may be achievable with substantially reduced nivolumab exposure.

### 3.7 Safety profile and toxicity management

Immune-related adverse events require structured clinical management. The development of CTCAE Grade 2 hypothyroidism in this patient represents a characteristic irAE associated with anti-PD-1 therapy, which has been correlated with favorable therapeutic outcomes across solid tumors (5, 10). The condition was managed with levothyroxine (150 µg daily) under endocrinological supervision without interrupting cancer treatment. The de-escalated intermittent dosing strategy avoided severe Grade 3/4 irAEs (such as pneumonitis or severe hepatitis) that frequently require treatment discontinuation (16, 17).

### 3.8 Limitations

Several limitations should be considered when interpreting this case report. First, as this was a single-patient observation, broad clinical conclusions regarding the general efficacy, safety, or optimal dosing of low-dose nivolumab cannot be drawn, and the findings require validation in prospective studies involving larger patient populations. Second, nivolumab was administered in combination with gemcitabine, which limits the ability to determine the independent therapeutic contribution of low-dose nivolumab to the observed clinical outcome. Third, comprehensive genomic profiling and tumor immune characterization were not performed beyond PD-L1 CPS assessment. Additional molecular biomarkers, including TMB and BRCA mutation status, as well as transcriptomic and TIL profiling, were not assessed because of financial constraints, thereby limiting mechanistic interpretation of the observed response. Finally, the proposed concept of a “minimum effective immune dose” remains a working hypothesis derived from available pharmacokinetic/pharmacodynamic literature and has not yet been prospectively validated. Further clinical and translational studies are therefore required to determine whether dose reduction can maintain therapeutic immune activity while preserving clinical efficacy.

## 4 Conclusion

In summary, combination therapy with gemcitabine chemotherapy and low-dose, intermittent nivolumab (40 mg fixed dose) achieved durable disease control (>22 months) with a manageable safety profile in a patient with high PD-L1-expressing mTNBC. The de-escalated dosing approach offered a pragmatic, potentially tolerable response to financial constraints and treatment toxicity while maintaining durable disease control in this patient. Prospective clinical studies evaluating dose-optimized, biomarker-driven ICI regimens are warranted to test this hypothesis and improve global treatment access in resource-constrained settings.

## Data Availability

The raw data supporting the conclusions of this article will be made available by the authors, without undue reservation.
